# Needle-Age Related Variability in Nitrogen, Mobile Carbohydrates, and δ^13^C within *Pinus koraiensis* Tree Crowns

**DOI:** 10.1371/journal.pone.0035076

**Published:** 2012-04-06

**Authors:** Cai-Feng Yan, Shi-Jie Han, Yu-Mei Zhou, Cun-Guo Wang, Guan-Hua Dai, Wen-Fa Xiao, Mai-He Li

**Affiliations:** 1 State Key Laboratory of Forest and Soil Ecology, Institute of Applied Ecology, Chinese Academy of Sciences, Shenyang, China; 2 Graduate University of Chinese Academy of Sciences, Beijing, China; 3 Research Station of Changbai Moutain Forest Ecosystems, Chinese Academy of Sciences, Erdaobaihe, China; 4 Key Laboratory of Forest Ecology and Environment, State Forestry Administration, Chinese Academy of Forestry, Beijing, China; 5 Tree Physioecology, Swiss Federal Research Institute WSL, Birmensdorf, Switzerland; Friedrich-Alexander-University Erlangen-Nurenberg, Germany

## Abstract

For both ecologists and physiologists, foliar physioecology as a function of spatially and temporally variable environmental factors such as sunlight exposure within a tree crown is important for understanding whole tree physiology and for predicting ecosystem carbon balance and productivity. Hence, we studied concentrations of nitrogen (N), non-structural carbohydrates (NSC = soluble sugars + starch), and δ^13^C in different-aged needles within *Pinus koraiensis* tree crowns, to understand the needle age- and crown position-related physiology, in order to test the hypothesis that concentrations of N, NSC, and δ^13^C are needle-age and crown position dependent (more light, more photosynthesis affecting N, NSC, and δ^13^C), and to develop an accurate sampling strategy. The present study indicated that the 1-yr-old needles had significantly higher concentration levels of mobile carbohydrates (both on a mass and an area basis) and N_area_ (on an area basis), as well as NSC-N ratios, but significantly lower levels of N_mass_ (on a mass basis) concentration and specific leaf area (SLA), compared to the current-year needles. Azimuthal (south-facing vs. north-facing crown side) effects were found to be significant on starch [both on a mass (ST_mass_) and an area basis (ST_area_)], δ^13^C values, and N_area_, with higher levels in needles on the S-facing crown side than the N-facing crown side. Needle N_mass_ concentrations significantly decreased but needle ST_mass_, ST_area_, and δ^13^C values significantly increased with increasing vertical crown levels. Our results suggest that the sun-exposed crown position related to photosynthetic activity and water availability affects starch accumulation and carbon isotope discrimination. Needle age associated with physiological activity plays an important role in determining carbon and nitrogen physiology. The present study indicates that across-scale sampling needs to carefully select tissue samples with equal age from a comparable crown position.

## Introduction

The crown of a tree is important because it contains foliage which captures light, photosynthesizes and provides energy for tree growth and reproduction. A tree’s crown also interacts with other atmospheric variables such as CO_2_, temperature, and humidity. Physioecology of leaves/needles in relation to temporal and spatial variability of those factors within a crown is of direct interest to ecologists and physiologists [1], [2], to understand the whole tree physiology and to predict the carbon balance and productivity at individual and ecosystem level.

A tree’s geometrical structure and foliar characteristics affect the solar radiation interception, leading to spatial heterogeneity of photosynthesis, growth, and biomass within a crown [3]–[5]. For example, net photosynthetic rates have been found to be higher on the S-facing crown side and at the top crown level than on the N-facing side and at the bottom crown level [6]–[8]. Peters et al. [8] reported that sun-exposed *Pinus canariensis* needles at the upper crown levels had higher net photosynthetic rates and stomatal conductance than needles which were shaded. Such effects have been explained as light effects, since more radiation should be intercepted on the sunlit crown side or at the upper crown levels, leading to significant crown position effects. Single leaf area, length and thickness, and stomatal parameters were also found to vary with crown position [9]–[13]. Significant azimuthal effects on both leaf thickness and density within *Fagus crenata* crown were also reported, with greater leaf thickness and density on the N-facing than the S-facing crown side [14].

Younger needles are located on the outer crown and older needles in the inner crown position within a tree’s crown. Hence, needle age within a conifer crown not only indicates needle age-dependent physiology but also implies a position effect [15]. Needle morphology and biochemistry were found to be needle-age dependent [8], [16]–[19]. Different aged needles of conifers showed different eco-physiological performance and, thus, may represent different levels of N and mobile carbohydrate concentrations. Li et al. [19] found that current-year needles had significantly lower concentrations of soluble sugars, starch, and NSC than 1-yr-old needles in *Pinus cembra* trees in July when the current-year needles were not fully mature. But at the end of the growing season, this difference disappeared [19]. The lower concentrations of mobile carbohydrates in current-year needles during the growing season was explained by dilution effects caused by lower density in non-mature organs [19], since younger needles had higher SLA (cm^2^g^−1^ as the ratio of projected needle area to needle dry mass) or lower leaf mass per unit leaf area (LMA) than older needles [9], [16].

Leaf δ^13^C values are closely correlated with chemical components and leaf morphology, such as leaf N [20] and starch concentration [21], and stomatal conductance [22]. Li et al. [18] demonstrated that δ^13^C in leaves was positively correlated with N and negatively correlated with SLA in *Quercus aquifolioides*. Values of δ^13^C have also been found to be associated with leaf or needle age [23], [24]. Barszczowska and Jedrysek [25] found more negative δ^13^C values in older foliage than in younger needles of *Pinus nigra* trees. Studies in several evergreen and deciduous species have also found less negative values of δ^13^C in younger leaves than in older leaves [26]–[28]. Other studies documented that δ^13^C values tended to be less negative at the upper canopy level compared to the lower canopy level [29]–[32]. For example, for the same-aged foliage across 10 tree species, δ^13^C values were significantly less negative in the upper canopy than in the lower canopy, and there were markedly differences in foliage δ^13^C values between the S- and N-facing crown side for 4 coniferous species but no such differences for 6 broad-leaved tree species [28].

*Pinus koraiensis*, a national protected plant species in China, is a dominant tree species of the climax vegetation (broad-leaved Korean pine mixed forest) in northeastern China. Recently, many national and international projects related to this species have been initiated, due to its economic and ecological importance (Han SJ, personal communication). These projects need more accurate sampling strategies to achieve the desired sample, in order to get comparable and comprehensive data across scales. To our knowledge, no studies have tested the temporal and spatial variations of N, mobile carbohydrates, and stable carbon isotope in different-aged needles within a *P. koraiensis* crown. Hence, we studied N, NSC, and δ^13^C within *P. koraiensis* crowns to understand needle age- and crown position-related physiology, in order to develop an accurate sampling strategy. We hypothesized that 1) concentrations of N, NSC (soluble sugars, starch), and δ^13^C are needle-age dependent, since the leaf morphology and physiological activity are age-dependent [7]–[10], [12], [18], [19], and 2) the concentrations of N, NSC, and δ^13^C in needles on the S-facing crown side or at the top crown level are higher than those on the N-facing side or at the bottom crown level, respectively, due to the differences in sunlight exposure within a crown [7]–[9], [14], [15].

## Materials and Methods

No specific permits were required for the described field studies. The relatively widely distributed species *P. koraiensis* has been protected in the study region, the present study related to conservation eco-physiology of this species did not need a special permit, and was carried out within the research area of our long-term forest ecosystem research station (Changbai Mountain Forest Research Station) established in 1979. On the other hand, the field sampling had no damage on the sample trees because we took only 20 grams of fresh needles for each sample.

### Study site and forest

The study forest, close to the Changbai Mountain Forest Ecosystem Research Station (127°47′E, 42°19′N, 738 m a.s.l.), ranged from 1000 to 1200 m a.s.l. on Changbai Mountain, Jilin Province, northeastern China. According to the climate data collected in the research station from 1970 to 2008, the mean annual temperature is 3.2°C, and the average temperature in January and July is −15.6°C and 19.7°C, respectively. The mean growing season temperature is 14.6°C (May to October). The mean annual precipitation ranges from 600 to 900 mm year^–1^, 70% of which falls between June and August. Soils were classified as mountain brown clay coniferous forest soil developed from lava. The forest is a naturally generated, mixed *Pinus koraiensis*-broadleaved forest dominated by *P. koraiensis*, *Tilia amurensis, Quercus mongolica, Fraxinus mandshurica* and *Acer mono* [33], [34].

### Sampling

Fifteen healthy *P. koraiensis* trees were randomly selected from the upper canopy layer (i.e. no stressed and shaded trees) within a 10 km^2^ area. Sample trees were 70–90 years old, 17.5–18.2 m in height and 30–50 cm in diameter at breast height. The length and width of crowns were 6–8 m and 5–7 m, respectively. The crown length of each sample tree was divided into 3 equal segments of top, middle, and bottom crown level.

New needles of *P. koraiensis* emerge in May at the beginning of the growing season and mature in summer (Zhou YM, personal communication), and the needle longevity is two years and rarely reaches 3 years in the study region. Li et al. [19] indicated that concentrations of carbohydrates in immature needles were instable. Given the large seasonal variation in mobile carbohydrates in *Pinus* species [19], [35], [36], we decided to take needle samples at the end of growing season, as suggested by Shi et al. [37], in order to have comparable samples of mature needles. From October 5 to 6, 2010, one leading branch was cut from the N-facing and S-facing crown side at the top, middle, and bottom crown level, respectively, using a pruning saw. A total of 6 branches were cut from each sample tree, climbed using climbing spikes strapped to the climber’s feet. Current-year and 1-yr-old needles were collected separately from the outer shoots in each branch cut. All samples were kept in a cool box and killed in a microwave oven (at the middle-high temperature for 45–60 seconds) and dried to constant weight at 65°C [38]. Dried needle materials were ground into fine powder (passed through 100 meshes) for analysis.

### Isotope analysis

The dried needle powder (approx. 0.2 mg) was put in a tin foil cup and then combusted in an elemental analyzer (Flash EA-1112, Carlo Erba Thermoquest, Italy) interfaced (Conflo II, Thermo Finnigan, Bremen, Germany) to a continuous-flow isotope ratio mass spectrometer (DELTA plus XL, Thermo Finnigan, Bremen, Germany).

Carbon isotope composition was reported by the following conventional δ^13^C values notation relative to Vienna Pee Dee Belemnite international standard [39]:

*δ* ^13^C(‰) * = * (*R_sa_/R_sd_* - 1) *×* 1000

where *R_sa_* and *R_sd_* are the molecular abundance ratios of carbon isotope (^13^C/^12^C) of the sample and the standard, respectively. The overall precision of the replicate samples measurements was estimated to be better than ±0.2‰ standard deviation.

### Measurement of specific leaf area

Twenty-five fresh needles from each sample were scanned (Founder Z1000, Founder Technology Group Inc., Beijing, China) at 600 dpi, and analyzed using public Scion Image software (Image 4.02 for windows, online available via http://www.scioncorp.com, National Institutes of Health, Bethesda MD, USA) to determine the projected needle area [40]. Afterwards the needle dry mass (70°C, 3 days) was determined and the SLA was calculated. SLA was then used to calculate concentrations of N and carbohydrates expressed on a projected needle area basis.

### Chemical analyses: total soluble sugars

The powdered leaf material (0.1 g) was put into a 10 ml centrifuge tube, where 5 ml of 80% ethanol were added. The mixture was incubated at 80°C in a water bath shaker for 30 min, and then centrifuged at 4000 rpm for 5 min. The pellets were extracted two more times with 80% ethanol. Supernatants were retained, combined, and stored at −20°C for soluble sugar determinations. The soluble sugar fraction was measured. Soluble sugars in the collected extracts were determined using the anthrone method [41]. An aliquot of the extract was hydrolysed in 5 ml of 0.4% anthrone solution (4 g anthrone in 1000 ml 95% H_2_SO_4_) in a boiling water bath for 15 min. After cooling, the sugar concentration was determined spectrophotometrically (ultraviolet-visible spectrophotometer 752S, Cany Precision Instruments Co., Ltd., Shanghai, China) at 620 nm. Glucose was used as a standard. The sugar concentration was calculated on a dry mass basis (SU_mass_, % d.m.) and a projected needle area basis (SU_area_, g m^–2^), respectively.

### Chemical analyses: starch

The ethanol-insoluble pellet was used for starch extraction. Ethanol was removed by evaporation. Starch in the residue was released in 2 ml distilled water for 15 min in a boiling water bath. After cooling to room temperature, 2 ml of 9.2 mol/L HClO_4_ were added. Starch was hydrolyzed for 15 min. Four ml distilled water was added to the samples. Samples were then centrifuged at 4000 rpm for 10 min. The pellets were extracted one more time with 2 ml of 4.6 mol/L HClO_4_. Supernatants were retained, combined, and filled to 20 ml. The starch concentration was measured spectrophotometrically (ultraviolet-visible spectrophotometer 752S) at 620 nm using anthrone reagent, and calculated by multiplying glucose concentrations by the conversion factor of 0.9. Glucose was used as a standard. The starch concentration was calculated on a dry mass basis (ST_mass_, % d.m.) and a projected needle area basis (ST_area_, g m^–2^), respectively.

### Chemical analyses: total nitrogen

The concentration of total N was determined in finely ground oven-dried samples by the micro Kjeldahl procedure, using CuSO_4_, K_2_SO_4_, and H_2_SO_4_ for digestion, and NH_3_ was determined on an auto-analyzer, using the indophenol-blue colorimetric method [42]. The N concentration was expressed both on a dry mass basis (N_mass_, % d.m.) and a projected needle area basis (N_area_, g m^–2^), respectively.

### Statistical analysis

All presented and discussed concentrations data, except where otherwise noted, are expressed on a dry mass basis (% d.m.). NSC is defined as the sum of the starch plus the total soluble sugars for each sample [19], [43]. NSC-N ratio is defined as the ratio of NSC concentration to N concentration for each sample [38], [44]. All data (NSC, starch, total soluble sugars, and N concentrations, δ^13^C, and SLA) were checked for normality by Kolmogorov–Smirnov-Tests. To test differences in the parameters mentioned above within a tree crown, three-factor ANOVAs were performed with needle age (current-year needles, and 1-yr-old needles), azimuthal direction (S-facing vs. N-facing crown side), and vertical crown level (top, middle, and bottom crown level) as factors, and followed, if significant, by one-factor ANOVAs to compare the means of those parameters within each factor (i.e. needle age, or azimuthal direction, or vertical crown level). To get a clear feature of the parameters studied within a tree crown, data were pooled, according to needle age, azimuthal direction, and vertical crown level, respectively, and the pooled data were analyzed using one-way ANOVAs. Pearson’s correlation coefficients were used to examine the relationships between variables. All statistical analyses were performed using SPSS 11.5 for windows.

## Results

### Needle-age effects

Needle age significantly affected the leaf total N and carbohydrates (NSC, soluble sugars, and starch) concentrations expressed both on a mass basis (Table 1) and on an area basis (data not shown), NSC-N ratios, and SLA, but not δ^13^C values (Table 1). The needle age effects seemed to be more pronounced at the middle- and bottom-crown rather than at the top-crown level (Table 2). Current-year needles had significantly lower N_area_ (−9.7%) but higher N_mass_ (+22.0%) and greater SLA (+34.7%) than 1-yr-old needles (Table 3). Conversely, both mass-based and area-based concentrations of NSC (−13.8% and −36.0%, respectively), sugars (−8.2%, −35.6%), and starch (−15.0%, −37.2%), as well as NSC-N ratio (−28.4%), reduced significantly in current-year needles compared to 1-year-old needles (Table 3). But the current-year needles and the 1-yr-old needles had the same sugar-starch ratio (∼4.15; Table 3).

**Table 1:** Effects of needle age, crown direction, and crown level on soluble sugars, starch, non-structural carbohydrates (NSC), and total nitrogen concentrations expressed on a dry mass basis, NSC-N ratio, δ^13^C, and SLA in *Pinus koraiensis* needles. F- and *P*-values are given (*n*  =  15).

		Nitrogen			Soluble sugars			Starch			NSC			δ^13^C			NSC-N ratio			SLA	
	*df*	F	*P*		F	*P*		F	*P*		F	*P*		F	*P*		F	*P*		F	*P*
Needle age (A)	1	27.485	**< 0.001**		3.930	**0.050**		17.977	**< 0.001**		6.227	**0.028**		1.268	0.282		25.268	**< 0.001**		22.051	**0.002**
Crown direction (D)	1	3.683	0.079		0.027	0.872		6.969	**0.022**		0.256	0.622		8.822	**0.012**		0.270	0.613		0.144	0.714
Crown level (L)	2	4.816	**0.029**		1.085	0.369		10.289	**0.002**		0.774	0.483		19.265	**< 0.001**		1.675	0.228		0.281	0.764
A×D	1	0.536	0.478		0.082	0.780		0.222	0.646		0.120	0.735		0.052	0.823		0.091	0.768		0.142	0.716
A×L	2	0.157	0.857		1.123	0.357		0.154	0.859		1.221	0.329		1.725	0.220		0.321	0.731		0.365	0.412
D×L	2	3.054	0.085		0.165	0.850		8.278	**0.006**		0.528	0.603		8.560	**0.005**		0.482	0.629		0.014	0.986
A×D×L	2	1.993	0.179		1.181	0.340		3.318	0.071		0.816	0.465		7.526	**0.008**		2.438	0.129		0.953	0.115

Note (1) significant differences (*P* < 0.05) are highlighted in bold, and (2) statistical analyses using data expressed on a projected area basis and on a dry mass basis showed the same results and were then not repeated.

**Table 2:** Mean values (±SE, *n* = 15) of concentrations (%, expressed on a dry mass basis) of soluble sugars, starch, non-structural carbohydrates (NSC), and nitrogen, NSC-N ratio, δ^13^C (‰), and SLA (cm^2^ g^–1^) in current year and one-year-old needles within *Pinus koraiensis* tree crown.

	Top crown level						Middle crown level						Bottom crown level				
	South			North			South			North			South			North	
	Current-yr needles	1-yr-old needles		Current-yr needles	1-yr-old needles		Current-yr needles	1-yr-old needles		Current-yr needles	1-yr-old needles		Current-yr needles	1-yr-old needles		Current-yr needles	1-yr-old needles
Nitrogen	1.16±0.03a	1.00±0.03b		1.15±0.09a	0.84±0.02b		1.20±0.07	0.97±0.00		1.21±0.10	1.06±0.01		1.48±0.20a	1.12±0.01b		1.15±0.01a	1.04±0.02b
Sugar	10.05±1.32	8.65±2.84		8.67±0.56	9.88±0.84		8.96±0.25b	11.92±0.19a		9.89±1.80	11.73±0.31		8.63±0.26b	11.97±1.49a		9.35±0.77	9.95±1.41
Starch	2.25±0.31b	2.96±0.01a		2.75±0.01	2.72±0.14		2.31±0.16	2.57±0.31		2.00±0.10b	2.67±0.01a		2.36±0.06b	2.72±0.05a		1.56±0.15	1.98±0.22
NSC	12.31±1.01	11.61±2.83		11.43±0.57	12.60±0.70		11.27±0.41b	14.49±0.11a		11.89±1.90b	14.41±0.32a		10.99±0.32b	14.70±1.55a		10.91±0.63	11.93±1.63
δ^13^C	−28.59±0.36	−28.27±0.05		−28.41±0.44	−29.27±0.40		−29.18±0.64	−28.62±0.15		−28.65±0.01	−28.63±0.21		−28.37±0.16a	−29.88±0.09b		−30.77±0.14	−30.35±0.32
NSC/N	10.64±0.64	11.60±2.55		9.96±0.29b	15.07±0.56a		9.44±0.89b	14.94±0.11a		10.05±2.36b	13.59±0.17a		7.59±1.24b	13.17±1.33a		9.53±0.59	11.55±1.74
SLA	83.52±5.73a	53.92±3.64b		79.80±3.10a	51.73±4.2b		84.25±2.61a	57.88±2.92b		82.34±2.22a	54.37±4.32b		76.42±5.13	74.54±4.37		82.01±2.65a	70.12±4.12b

Note (1) different letters indicated statistically significant (*P* < 0.05) difference in each parameter between current-year needles and one-year-old needles within each crown position (no letters  =  no statistical difference), and (2) analyses using data expressed on a projected area basis and on a dry mass basis showed the same statistical results between current-yr and 1-yr-old needles within each category, and were then not repeated.

**Table 3:** Mean values (*n* = 15) of concentrations of needle soluble sugars, starch, non-structural carbohydrates (NSC), and total nitrogen expressed on a dry mass basis (dM-based, % d.m.) and on a projected area basis (pA-based, g m^–2^), as well as NSC-N ratio, δ^13^C content values (‰), and SLA (cm^2^ g^–1^) in needles within *Pinus koraiensis* tree crown.

	Nitrogen			Sugars			Starch			NSC			NSC-N ratio	Sugar-starch ratio	δ^13^C	SLA
	dM-based	pA-based		dM-based	pA-based		dM-based	pA-based		dM-based	pA-based					
**Needle age**																
Current-year	1.22a	1.50b		9.26b	11.38b		2.21b	2.72b		11.46b	14.08b		9.54b	4.19	−28.99	81.39a
1-year-old	1.00b	1.66a		10.68a	17.68a		2.60a	4.30a		13.29a	21.99a		13.32a	4.11	−29.18	60.42b
**Azimuthal direction**																
South	1.15	1.64a		10.03	14.32		2.52a	3.60a		12.56	17.93		11.23	3.98	−28.83a	70.06
North	1.07	1.49b		9.91	13.81		2.28b	3.18b		12.19	16.99		11.62	4.35	−29.34b	71.75
**Vertical crown level**																
Top	1.03b	1.53		9.31	13.85		2.67a	3.97a		11.99	17.83		11.82	3.49	−28.63a	67.24
Middle	1.11ab	1.59		10.63	15.25		2.39ab	3.43b		13.01	18.66		12.00	4.45	−28.79a	69.71
Bottom	1.19a	1.57		9.97	13.16		2.15b	2.84c		12.13	16.01		10.46	4.64	−29.84b	75.77

Different letters indicate statistically significant (*P* < 0.05) difference within column for each category (no letters  =  no statistical difference).

### Azimuthal effects

Effects of crown directions were found to be significant on starch and δ^13^C values in needles (Table 1), but these effects were dependent upon the crown-levels (crown direction x crown-level interaction for both starch and δ^13^C with *p* < 0.01) (Table 1). The crown direction effects seem to be more pronounced at the lower crown levels rather than at the upper crown levels (Table 2). Needles on the S-facing crown side had significantly higher starch concentrations (+9.5% for ST_mass_, +11.7% for ST_area_) and higher δ^13^C values (−28.83‰ vs. −29.34‰) compared to needles on the N-facing crown side (Table 3). Not N_mass_ but N_area_ showed significantly lower levels in needles on the N-facing crown side (−9.2%) than on the S-facing crown side (Table 3). Needle SLA did not significantly vary with crown directions (Table 1) but tended to have lower level in the sun-exposure crown side (Table 3).

### Effects of vertical crown level

Increasing crown levels significantly affected needle δ^13^C values, starch, and N_mass_ concentration (Table 1) but not N_area_ (statistical data not shown; see also Table 3). The crown level effects on needle starch and δ^13^C were found to be dependent upon the crown-direction (e.g. crown direction x crown level interaction for both starch and δ^13^C with *p*<0.01; Table 1). Needle N_area_ did not change, but N_mass_ concentrations significantly decreased, and needle ST_mass_, ST_area_, and δ^13^C values significantly increased with increasing crown level (Table 3). Although concentrations of sugars and NSC did not change, the sugar-starch ratio decreased with increasing crown level from 4.64 (bottom), to 4.45 (middle) and 3.49 (top crown level) (Table 3). Needle SLA did not significantly vary with crown levels (Table 1) but tended to decrease with increasing crown levels (Table 3).

## Discussion

### Variations in needle N concentration and SLA within a tree crown

The present study found that the total N_mass_ concentration was significantly higher in current-year needles than in 1-yr-old needles within a crown (Table 3). Leaf age-effects on foliage N concentration have already been reported for several coniferous [18], [45]–[49] and broad-leaved species [50], [51]. Needle N_mass_ declined steadily with leaf age for both *Pinus aristata* and *Pinus contorta* [45] and *Pinus sylvestries* [46]. The same age-related results of N_mass_ were also reported for other different coniferous species [47]–[49]. For broad-leaved species, Tateno and Kawaguchi [50] found that foliage N_mass_ concentrations decreased with increasing leaf age in both *Podocarpus nagi* and *Neolitsea aciculate*, and Mickelbart [51] observed that older leaves had higher N_mass_ than younger leaves in *Acer*×*freemanii* trees. These results may suggest that the contents of mobile nutrients (e.g. N) in the younger needles/leaves represent both N uptake and re-translocation form older foliages since the younger foliages are physiologically more active than the older ones.

Total N_mass_ concentrations did not differ between needles on the S-facing crown side and N-facing crown side (Table 3). Similarly, Mickelbart [51] also found that foliage N_mass_ was slightly higher but not statistically different in leaves on the S-facing crown side compared to the N- and W-facing crown side of *Acer*×*freemanii* trees. But Perica [52] reported that leaf N_mass_ concentrations were significantly higher (+11.8%, *p*<0.001) on the S-facing crown side than on the N-facing crown side in olive (*Olea europaea* L.) trees.

Needle N_mass_ concentrations decreased significantly with increasing vertical levels within a crown (Table 3). Previous studies documented an increase [45], [53], a decrease [54], [55], or no change [13], [30] in leaf N concentrations with increasing crown levels. For example, Nippert and Marshall [53] found that the mean N_mass_ was significantly (*p*<0.0001) higher in sun foliage than in shade foliage in *Abies grandis* and *Pseudotsuga menziesii* var. *glauca* throughout the growing season. Foliar N_area_ was significantly greater in the top third than in the bottom third of the crown for *Pinus contorta* ssp. *latifolia* [56]. But Dale and Causton [55] found that higher N_mass_ concentration occurred in shaded conditions rather than in higher light conditions for *Veronica* spp. because N uptake in shaded conditions exceeded its metabolic requirement. Han et al. [57] found that needle N_area_ concentration decreased by 22–27% from the upper to the lower crown level, as well as from the outer crown to the inner crown, corresponding to the decrease in photosynthetic photon flux density, in *Pinus densiflora* trees. Livingston et al. [30] found that there were poor relationships between N concentration allocation and intercepted radiation in young *Pinus radiata* trees grown in a plantation in New Zealand.

However, our results indicated that the effects of needle age or crown position on N depended upon whether N concentration was expressed on a mass basis or an area basis (Table 3). McGarvey et al. [58] found that not N_mass_ but N_area_ increased significantly with increasing vertical crown levels in *Pinus taeda* and *Pinus elliottii* var. *elliottii*. Griffin [59] observed that leaf N_mass_ significantly decreased by 17.5% (4.0%, 3.8%, and 3.3% for foliage at lower, middle, and upper canopy, respectively), but leaf N_area_ significantly increased (2.6, 2.7, and 3.0 g m^–2^ for foliage at lower, middle, and upper canopy, respectively) from lower canopy leaves to upper canopy leaves in *Populus deltoides* trees.

In line with previous findings gained from different conifers [19], [60], [61], we found that needle N concentration was positively correlated with SLA (*R^2^*  =  0.741, *p* < 0.01) (Table 4), and that SLA decreased significantly with increasing needle age (Table 3). Similar age effects on needle SLA have been widely reported for *Pinus* species [17], [62] and other coniferous species [17]. Compared to current-year needles with greater SLA, older needles with lower SLA (i.e. higher LMA) contained more structural compounds (e.g. lignin), which diluted needle total N concentration expressed on a dry mass basis [19], [63]. Niinemets [47] indicated that LMA of *Picea abies* needles increased with increasing needle age due to greater needle density, thickness and width in older needles.

**Table 4:** Correlation coefficients among NSC (non-structural carbohydrates), soluble sugars, starch, nitrogen contents, NSC-N ratio, δ^13^C contents, and SLA for *Pinus koraiensis* needles.

	Nitrogen	Soluble Sugars	Starch	NSC	δ^13^C	NSC-N ratio	SLA
Nitrogen	1	−0.262	−0.333	−0.326	0.170	−0.765**	0.741**
Soluble Sugars		1	0.093	0.973**	−0.170	0.771**	−0.381
Starch			1	0.318	0.533**	0.424*	−0.625*
NSC				1	−0.039	0.832**	−0.488
δ^13^C					1	−0.084	−0.223
NSC-N ratio						1	−0.763**
SLA							1

* *P*<0.05.

** *P*<0.01.

Leaves exposed to high light at the higher crown level tended to have lower SLA (Table 3). Decreases in needle SLA with increasing crown level have already been widely observed in *Pinus* and other coniferous species [58], [64]–[66]. Increases in SLA with canopy depth (Table 3) may be a mechanism of needles to capture more light per unit leaf mass at lower irradiances [67]. Previous studies also proposed that water stress and hydraulic limitations may be two main reasons to influence needle morphology, resulting in smaller thicker needles in the upper crown of large old trees [1], [65], [66]. Lower needle SLA on the S-facing crown side and at the higher crown level found in the present paper may be a result of starch accumulation in the sun-exposed needles (Table 3).

Leaf N concentration was found to be strongly positively correlated with leaf photosynthetic capacity both in tropical and temperate forest trees [58], [64], [68], [69]. Schoettle [45] found that current-year needles had higher N concentration and higher photosynthetic capacity than 1-yr-old needles in adult *Pinus aristata* trees. A strongly positive relationship between foliar N_mass_ and light-saturated photosynthesis rates (A_max_) was reported for *Pinus taeda* trees [58]. Leaves having higher photosynthetic rate may produce sugar more efficiently.

### Variations in mobile carbohydrates within a tree crown

Our study showed that concentrations of NSC, soluble sugars, and starch were higher in 1-yr-old needles than in current-year needles (Table 3). This finding is consistent with the results gained from *Pinus cembra* [19]. According to Niinemets [47], older needles have higher abilities to capture light and accumulate NSC. However, Li et al. [5] found that needle NSC concentrations increased with needle age for younger needles (<2 years old) and decreased with needle age for older needles (>2 years old) in *Abies georgei* trees. Concentrations of sugars and NSC in needles did not differ with azimuthal direction between the S- and N-facing crown side (Table 3) and also did not vary with increasing crown level (Table 3). Similar results gained from *Pinus cambra* trees have been reported [19]. Li et al. [5] did not detect any crown position effects on needle NSC in *Abies georgei* and in *Juniperus saltuaria* grown in Tibetan Plateau**.** Würth et al. [70] also did not find any significant differences in NSC concentration in leaves between sun and shade positions in 17 tropical tree species ranging from 75 to150 years old in Panama. These findings [5], [70], together with the present results (see Table 3), indicated that the effects of sunlight exposure (S-facing vs. N-facing crown side, and top, middle, and bottom crown level) within a crown play only negligible role in determining NSC and soluble sugars in needles or leaves. According to Hoch and Körne [71], levels of mobile carbohydrates in needles/leaves may be mainly affected by low temperature.

Levels of NSC concentrations were determined by soluble sugars (*p* < 0.01) rather than by starch (Table 4). Not soluble sugars and NSC but starch concentrations in needles significantly increased with sun exposure (Table 3). This result may be explained as a result of (1) light-induced increases in photosynthesis [56], [57] and (2) sucrose-starch conversion and partitioning changed by excess photosynthetic capacity over respiration and other use with increasing crown level associated with sun exposure. The light-saturated photosynthetic rate was found to be greater for needles in the upper crown than for needles in either the middle or lower crown locations for different *Pinus* species [56], [57]. Excess sugars are rapidly converted to starch temporarily stored in the leaves. Insufficient photosynthesis (e.g. lower light intensity or other disturbance) leads to conversion of starch to sugars to meet the needs of a plant. These also indirectly explained why the sugar-starch ratio decreased steadily with increasing crown level (Table 3).

The present study showed that the NSC-N ratio was significantly higher in 1-yr-old needles (+40.8%) compared to current-year needles (Table 3). Similarly, Li et al. [18] found that old tissues had higher C-N ratios than younger ones in *Quercus aquifolioides* in SW China. In contrast, Griffin et al. [59] showed significant decrease in C-N ratios from upper canopy to lower canopy leaves in *Populus deltoides*.

### Foliage δ^13^C within a tree crown

Barszczowska and Jedrysek [25] reported that δ^13^C values were higher (less negative) in younger needles (2 to 7 months old) than in older needles (1 to 2 years old) in *Pinus nigra* trees in Croatia and southern Spain. Würth et al. [72] and Holtum and Winter [73] also found that δ^13^C values in young leaves of several tropical tree species were less negative than those in older leaves in Panama throughout a seasonal cycle. Our results showed that the δ^13^C value in current-yr needles did not differ with that in 1-yr old needles (Table 3). This result may be caused by a combined effect of positive effects of starch and negative effects of sugars and NSC on δ^13^C (Table 4). In line with the present study, Gebauer and Schulze [74] found that the δ^13^C values of needles (*Picea abies*) did not change consistently with needle age, but did decrease from the sun- to the shade-crown.

Crown position including azimuthal direction and vertical crown level significantly affected needle δ^13^C values in our study (Table 3), which is consistent with some previous studies [75], [76]. Holtum and Winter [73] investigated 9 tree species in tropical forests and found that canopy position significantly influenced leaf δ^13^C values. A lower rate of photosynthesis in shaded needles, due to light limitation, might result in more negative δ^13^C values at lower crown levels [29]. Another possible explanation is the hydraulic limitation hypothesis [63], [69], [77] that expects decreasing water potential and increasing water stress in foliage at upper crown level, leading to declines in carbon isotope discrimination (less negative δ^13^C values) [32]. For example, Ishii et al. [78] found that bulk leaf water potential (*Ψ*) decreased and δ^13^C content values increased with increasing crown level in *Sequoia sempervirens* trees in California, USA. Less negative δ^13^C may be associated with higher stomatal conductance and higher photosynthetic capacity in the upper canopy compared to the lower canopy, as has been observed for different *Pinus* species [56], [57]. This trend could also be caused by the accumulation of starch in the needles at the higher crown level (Table 3). Our results showed that δ^13^C values were not correlated with the concentrations of NSC and soluble sugars, but significantly positively correlated with the starch concentration (*R* ^2^ = 0.533, *p*<0.01, Table 4). In line with our results, Jäggi et al. [21] pointed out that needle δ^13^C value had a strong correlation with starch concentration in *Picea abies* trees in the Swiss Plateau.

### Conclusion

The present study indicated that needle age had significant effects on nitrogen and carbon physiology. Azimuthal (S-facing vs. N-facing crown side) effects were found to be significant on starch and δ^13^C values with higher levels in needles on the S-facing crown side compared to the N-facing crown side. Needle N_mass_ significantly decreased, but needle starch and δ^13^C significantly increased with increasing vertical crown levels. Previous studies have reported marked age effects and effects of crown position associated with sunlight exposure on leaf physioecological performance [19], [57], [79]. Our results suggest that the sun-exposed crown position related to photosynthetic activity and water availability affects starch accumulation and carbon isotope discrimination. Needle age associated with physiological activity plays an important role in determining carbon and nitrogen physiology. The present study indicates that across-scale sampling needs to adhere to a strict age specific tissue selection from a comparable crown position.
